# Digital health literacy as a super determinant of health: More than simply the sum of its parts

**DOI:** 10.1016/j.invent.2022.100500

**Published:** 2022-02-07

**Authors:** Robin van Kessel, Brian Li Han Wong, Timo Clemens, Helmut Brand

**Affiliations:** a Department of International Health, Care and Public Health Research Institute (CAPHRI), Faculty of Health, Medicine and Life Sciences, Maastricht University, the Netherlands; b Studio Europa, Maastricht University, the Netherlands; c Research Committee, Global Health Workforce Network (GHWN) Youth Hub, World Health Organization, Switzerland; d The *Lancet* and Financial Times Commission on Governing Health Futures 2030: Growing up in a digital world, Global Health Centre, The Graduate Institute, Geneva, Switzerland; e Digital Health Section, European Public Health Association (EUPHA), Utrecht, the Netherlands; f Prasanna School of Public Health, Manipal Academy of Higher Education, Manipal, Karna-taka, India

## Abstract

• Civic literacy refers to the ability to engage meaningfully with one's community.
• Digital, health, and civic literacy are key predictors for digital health literacy.
• The extent to which these three affect digital health literacy remains unclear.
• Building digital health literacy is vital to limit inequalities from expanding.

Digital media has permeated all strata of daily life to the point where people engage with them for several hours each day on average. While its relevance for health-related purposes is constantly increasing, digital media can simultaneously play a draconic role in the spread of factually incorrect information; this not only sows doubt but can also be detrimental to individual and public health (de Albuquerque Veloso Machado et al., 2021). To harness the full potential of digital media to support health and well-being as well as to mitigate or counteract the effects of mis- and disinformation, three fundamental skills should be continuously developed: digital literacy, health literacy, and digital health literacy.

Digital literacy is described as “the ability to use information and communication technologies to find, evaluate, create, and communicate information, requiring both cognitive and technical skills” (American Library Association, 2017; UNESCO (United Nations Educational Scientific and Cultural Organization), 2011). This definition is constantly developing as digital transformations and applications grow more potent and complex. Nowadays, digital literacy is becoming increasingly important to the point where it can be regarded as a fundamental prerequisite for meaningfully participating in modern society (Sieck et al., 2021; Scheerder et al., 2017). Health literacy – the ability to obtain, read, understand, and use health-care information to make appropriate/informed health decisions (Sørensen et al., 2012; Sørensen et al., 2015) – is increasingly becoming a core skill for health-related information on the Internet (Lwin et al., 2020). Digital health literacy, at first glance, can be regarded as the convergence of digital literacy and health literacy (Honeyman et al., 2020). It is also important to consider that these factors likely covary to a certain extent. Age, sex, socioeconomic status (i.e. income, employment, and education), health status, and living in urban versus rural environments are all factors that can influence the development of both health and digital literacy (Scheerder et al., 2017; Sørensen et al., 2012; Sørensen et al., 2015; Honeyman et al., 2020; Odone et al., 2019; Kickbusch et al., 2021).

Both health and digital literacy are commonly conceptualised through competency-based frameworks. Health literacy is elaborately expressed through a matrix of four dimensions (access/obtain information relevant to health, understand information relevant to health, process/appraise information relevant to health, and apply/use information relevant to health) that are applied across three domains (healthcare, disease prevention, and health promotion) (Sørensen et al., 2012). It has also been described as a “social vaccine” amidst the COVID-19 pandemic that it enables individuals and communities to positively skew the spread of disease by finding and applying information related to the virus (Okan et al., 2022). A European Commission framework on digital competencies takes a similar approach to digital literacy by depicting five dimensions (information and data literacy, communication and collaboration, digital content creation, safety, and problem-solving), each with four to six sub-dimensions that illustrate a core competence of digital literacy (Joint Research Centre (European Commission) et al., 2017). These frameworks showcase the complexity and multidimensionality of health and digital literacy and therefore highlight the need to conceptualise digital health literacy in the context of a competence framework. The Transactional Model of eHealth Literacy outlines four competence levels of digital health literacy (Paige et al., 2018):

(1) functional: the ability to successfully read and write about health using technological devices;
(2) communicative: the ability to control, adapt, and collaborate communication about health with others in online social environments;
(3) critical: the ability to evaluate the relevance, trustworthiness, and risks of sharing and receiving health-related information through the digital ecosystem (e.g. the Internet); and
(4) translational: the ability to apply health-related information from the digital ecosystem (e.g. the Internet) in different contexts.

Even though digital and health literacy are related to digital health literacy, the reality is likely more complex. The relationship between digital, health, and digital health literacy is a multi-dimensional one where each competence domain of digital and health literacy may affect one or more competence domains of digital health literacy (Paige et al., 2018; Risling, 2019), but certain competencies of digital health literacy may not be covered by neither digital literacy nor health literacy (Kickbusch et al., 2021).

Civic literacy – the knowledge and ability to participate in one's society and community – has recently been regarded as a novel digital determinant of health (Kickbusch et al., 2021). Digital health services vary depending on how much citizen/patient/consumer input they require. For instance, wearables and tracking devices require little-to-no input; whereas accessing virtual healthcare or telemedicine requires substantially more input. In other words, the early entry points into digital health (wearables and other tracking devices, but also electronic health records) did not require conscious citizen input. As digital health services increasingly develop and start to compete with traditional health services (Fernandez et al., 2021), the skills that a citizen requires to fully benefit from digital health services grow more complex as well.

Another element to consider when operationalising digital health literacy is how it is perceived in different cultural settings. Reasons to believe such differences in cultural interpretations exist are found in a recent report by the Health Foundation and Framework Institute (Elwell-Sutton et al., 2019). Their study in the United Kingdom highlights that individual behaviours and choices and access to affordable healthcare dominate the public perception of what factors affect an individual's health and that 24% of the UK population believes health is fully the responsibility of the individual. They subsequently identify eight cultural models of how health can be conceptualised, which can be categorised as a mix of two archetypes: an individualistic approach and an ecological approach. It is then concluded that the general population typically adopts a more individualistic approach to health, whereas health professionals (especially those working on the social determinants of health) take a more ecological stance towards health. It is, therefore, safe to assume that – if an effort is being placed on educating the public about the social determinants that can affect health (e.g. in primary or secondary education) – the perception of health and who is responsible for ensuring it will change.

During the launch of The Lancet and Financial Times Commission on Governing Health Futures 2030: Growing up in a digital world at the 2021 World Health Summit, Marelize Gorgens of the World Bank emphasised that “we need to build a larger demand for digital health services, as they are not second-tier forms of healthcare, but first-tier of a different, digital nature” (World Health Summit, 2021). Seeking analogue health services is also entrenched in the habits of the general public, while digital services are often simply not considered – either due to lack of awareness or lack of trust. Both may be ameliorated by improving digital health literacy (Kickbusch et al., 2021), furthering the need to understand how digital, health, civic, and digital health literacy interact in the wider context of the social determinants of health.

Digital health literacy and internet connectivity have recently been acknowledged as “super social determinants of health” in that they have implications for the wider social determinants of health (Sieck et al., 2021). However, one must possess the requisite civic, digital, and health literacies in order to meaningfully participate in a digital era and achieve optimal health and well-being (Honeyman et al., 2020; Kickbusch et al., 2021; van Kessel et al., 2022a). As such, a framework is needed to clarify what exactly digital health literacy comprises in relation to its structural building blocks and how these building blocks are interlinked in a digital world (Wong et al., 2022). Ultimately, while digital transformations have tremendous potential to benefit public and population health, they are equally capable of exacerbating existing inequalities (van Kessel et al., 2022b). Conceptualising and building digital health literacy is therefore not only necessary at the professional level (who can develop, deploy, recommend, and prescribe the use of digital health services), but also at the public level (who will make up the user-base of digital health services). Social and cultural determinants can heavily affect the way digital health literacy is built up. Having a clear model for the determinants of digital health literacy in place is key to not only frame digital health literacy as a set of core competencies but also contextualise it amidst health literacy, digital literacy, civic literacy, and social and cultural determinants.

## Funding

No funding was acquired for this article.

## Data availability

All data was openly available. No data was generated for the purpose of this article.

## CRediT authorship contribution statement

RVK: conceptualisation, data interpretation, writing the original draft and editing, visualisation, and supervision.

BLHW: conceptualisation, data interpretation, and writing the original draft and editing.

TC: Writing the original draft and editing.

HB: Writing the original draft and editing.

## Declaration of competing interest

The authors declare that they have no known competing financial interests or personal relationships that could have appeared to influence the work reported in this paper.

## References

[bb0005] American Library Association (2017). https://literacy.ala.org/digital-literacy/.

[bb0010] de Albuquerque Veloso Machado M., Roberts B., BLH Wong, van Kessel R., Mossialos E. (2021). The relationship between the COVID-19 pandemic and vaccine hesitancy: a scoping review of literature until August 2021. Front. Public Health.

[bb0015] Elwell-Sutton Tim, Marshall Louise, Bibby Jo, Volmert Andrew (2019). https://www.health.org.uk/sites/default/files/upload/publications/2019/Reframing%20the%20conversation%20on%20social%20determinants.pdf.

[bb0020] Fernandez E., Woldgabreal Y., Day A., Pham T., Gleich B., Aboujaoude E. (2021). Live psychotherapy by video versus in-person: a meta-analysis of efficacy and its relationship to types and targets of treatment. Clin. Psychol. Psychother..

[bb0025] Honeyman M., Maguire D., Evans H., Davies A. (2020). Digital Technology and Health Inequalities: A Scoping Review [Internet].

[bb0030] Carretero S., Vuorikari R., Punie Y., den Brande L.V., Joint Research Centre (European Commission) (2017). https://data.europa.eu/doi/10.2791/11517.

[bb0035] Kickbusch I., Piselli D., Agrawal A., Balicer R., Banner O., Adelhardt M., Capobianco E., Fabian C., Singh Gill A., Lupton D., Medhora R.P., Ndili N., Ryś A., Sambuli N., Settle D., Swaminathan S., Morales J.V., Wolpert M., Wyckoff A.W., Xue L., Bytyqi A., Franz C., Gray W., Holly L., Neumann M., Panda L., Smith R.D., Georges Stevens E.A., Wong B.L.H. (2021). The lancet and financial times commission on governing health futures 2030: growing up in a digital world. Lancet.

[bb0040] Lwin M.O., Panchapakesan C., Sheldenkar A., Calvert G.A., LKS Lim, Lu J. (2020). Determinants of eHealth Literacy among Adults in China. J Health Commun.

[bb0045] Odone A., Buttigieg S., Ricciardi W., Azzopardi-Muscat N., Staines A. (2019). Public health digitalization in Europe. Eur. J. Public Health.

[bb0050] Okan O., Messer M., Levin-Zamir D., Paakkari L., Sørensen K. (2022). Health literacy as a social vaccine in the COVID-19 pandemic. Health Promot Int.

[bb0055] Paige S.R., Stellefson M., Krieger J.L., Anderson-Lewis C., Cheong J., Stopka C. (2018). Proposing a transactional model of ehealth literacy: concept analysis. J Med Internet Res.

[bb0060] Risling T. (2019). Beyond the Divide: Exploring the Digital Determinants of Health. Can Health Infoway Infoway Partnersh Conf [Internet]. https://www.infoway-inforoute.ca/en/component/edocman/supporting-documents/partnership/3770-ipc2019-day02-1040-1110-tracie-risling-en-pdf?Itemid=101.

[bb0065] Scheerder A., van Deursen A., van Dijk J. (2017). Determinants of Internet skills, uses and outcomes. A systematic review of the second- and third-level digital divide. Telematics Inform..

[bb0070] Sieck C.J., Sheon A., Ancker J.S., Castek J., Callahan B., Siefer A. (2021). Digital inclusion as a social determinant of health. Npj Digit Med.

[bb0075] Sørensen K., Van den Broucke S., Fullam J., Doyle G., Pelikan J., Slonska Z., Brand H., (HLS-EU) Consortium Health Literacy Project European (2012). Health literacy and public health: a systematic review and integration of definitions and models. BMC Public Health.

[bb0080] Sørensen K., Pelikan J.M., Röthlin F., Ganahl K., Slonska Z., Doyle G., Fullam J., Kondilis B., Agrafiotis D., Uiters E., Falcon M., Mensing M., Tchamov K., Brand H., van den Broucke S., on behalf of the HLS-EU Consortium (2015). Health literacy in Europe: comparative results of the European health literacy survey (HLS-EU). Eur J Public Health.

[bb0085] UNESCO (United Nations Educational Scientific and Cultural Organization) (2011). Digital literacy in education [Internet]. https://iite.unesco.org/pics/publications/en/files/3214688.pdf.

[bb0090] van Kessel R., BLH Wong, Rubinić I., O’Nuallain E., Czabanowska K. (2022). Is Europe prepared to go digital? Making the case for developing digital capacity: an exploratory analysis of Eurostat survey data. PLOS Digit Health.

[bb0095] van Kessel R., Hrzic R., O’Nuallain E., Weir E., Wong B.L.H., Anderson M., Baron-Cohen S., Mossialos E. (2022). The digital health paradox: international policy perspectives to address the increased health inequalities for people living with disabilities. J. Med. Internet Res..

[bb0505] Wong B.L.H., Maaß L., Vodden A., van Kessel R., Sorbello S., Buttigieg S., Odone A. (2022). The dawn of digital public health in Europe: implications for public health policy and practice. The Lancet Regional Health - Europe.

[bb0100] World Health Summit (2021). https://www.youtube.com/watch?v=Bm9kHdoi3q4.

